# Watching Living Cells in Action in the Exocrine Pancreas: The Palade Prize Lecture

**DOI:** 10.1093/function/zqac061

**Published:** 2022-12-08

**Authors:** Ole H Petersen

**Affiliations:** School of Biosciences, Sir Martin Evans Building, Cardiff University, Wales, CF10 3AX, UK

**Keywords:** imaging, live morphology, calcium signaling, secretion, exocrine pancreas, acute pancreatitis, acinar cell, stellate cell, pancreatic immune cell

## Abstract

George Palade’s pioneering electron microscopical studies of the pancreatic acinar cell revealed the intracellular secretory pathway from the rough endoplasmic reticulum at the base of the cell to the zymogen granules in the apical region. Palade also described for the first time the final stage of exocytotic enzyme secretion into the acinar lumen. The contemporary studies of the mechanism by which secretion is acutely controlled, and how the pancreas is destroyed in the disease acute pancreatitis, rely on monitoring molecular events in the various identified pancreatic cell types in the living pancreas. These studies have been carried out with the help of high-resolution fluorescence recordings, often in conjunction with patch clamp current measurements. In such studies we have gained much detailed information about the regulatory events in the exocrine pancreas in health as well as disease, and new therapeutic opportunities have been revealed.

## George E Palade

The International Association of Pancreatology’s (IAP) Palade Prize is named after George E Palade, who shared the 1974 Nobel Prize in Physiology or Medicine with Albert Claude and Christian de Duve “for their discoveries concerning the structural and functional organization of the cell.” One of Palade’s many great achievements was the elucidation of the secretory pathway in pancreatic acinar cells, including the discovery of exocytosis. This was the central theme for his Nobel Prize Lecture.^1^

**Figure 1.**
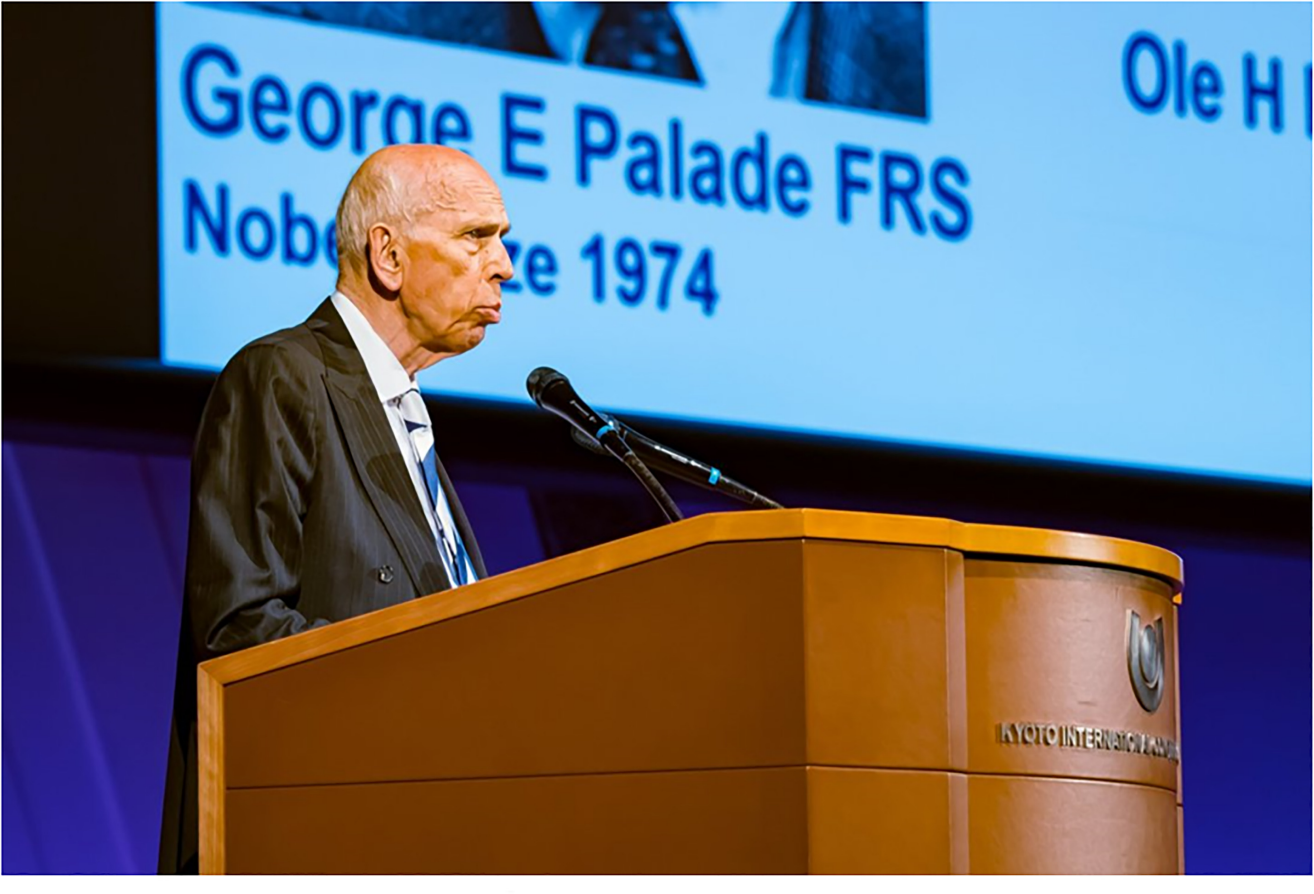
Ole Petersen presenting the 2022 George E Palade Prize Lecture at the International Conference Center in Kyoto, Japan on 7th July 2022.

It was a great honor for me to receive the Palade Prize and to deliver the Palade Prize Lecture on 7th July 2022, at the Joint meeting of the Japan Pancreas Society with the IAP, at Kyoto’s International Conference Center (Figure 1). I was particularly happy to receive this award, because I had the privilege of interacting with Palade at several cell biology meetings in the 1970s and in the 1980s as well as during visits to his department at Yale University. Furthermore, I was happy that Palade had cited one of my early papers^2^ in his published Nobel Prize Lecture.^1^ In this paper,^2^ I had shown that the two physiological stimulants of exocrine pancreatic secretion, namely acetylcholine (ACh) and cholecystokinin (CCK), depolarize the acinar cell membrane and I had provided some (indirect) evidence that this was associated with a rise in the cytosolic Ca^2+^ concentration ([Ca^2+^]_i_). In conjunction with a further paper^3^ from my sabbatical year at the University of Cambridge, in collaboration with John A Williams (a former Palade Prize winner), this provided the earliest evidence indicating that exocrine pancreatic secretion is acutely controlled by changes in [Ca^2+^]_i_.

Although much of Palade’s work was focused on the exocrine pancreas, it was highly relevant to cell biology in general. In fact, Palade was interested in understanding general cell properties, but had chosen the acinar cell of the pancreas as his study object because of its special characteristics. These included: the polarized structure of the acinar cells—enabling a clear spatial separation of the different elements of the secretory pathway, its large endoplasmic reticulum (ER) and the fact that the pancreatic acinar cell produces large quantities of protein. It was also important for Palade that previous work on the exocrine pancreas had led to work of general significance.^1^ Secretin, the first hormone to be discovered,^4^ stimulates a bicarbonate-rich fluid secretion from the pancreatic duct cells. The first preparation of pure enzymes, including trypsin and chymotrypsin from the pancreas, was made by John Northrop, who received the Nobel Prize for this work.^5^

Palade looked for mechanisms that were universally important and believed that nature uses such mechanisms in many different contexts. From my discussions with Palade, it was clear to me that he believed that exocytosis was universally activated by Ca^2+^. He felt that the well-established mechanism in nerve endings, namely that depolarization of the membrane opens Ca^2+^ channels,^6^ was likely to work everywhere, including in the exocrine glands. That a rise in [Ca^2+^]_i_ is the principal activator of secretion, also in exocrine glands, is now well established, although the mechanism is different from what Palade thought, as discussed later in this article. However, there are exceptions. Palade had difficulties acknowledging the results of Michael Schramm and Zvi Selinger showing that in the parotid gland the main intracellular messenger activating exocytotic secretion is cyclic AMP.^7^ In this context, I witnessed clashes between Palade and Schramm at several conferences. Palade’s opinion that the depolarization-elicited Ca^2+^ influx is the activator of pancreatic secretion, undoubtedly influenced his collaborator George Scheele, who published experiments that were interpreted to confirm this view.^8,9^ This turned out to be wrong, as I shall discuss below in the section on physiological Ca^2+^ signals.

Palade’s selection of the exocrine pancreas as his principal study object, was a lucky choice and paid off handsomely. He pioneered electron microscopical studies and enhanced them ingeniously with the so-called pulse-chase technique.^1,10,11^ My own primary interest was the acute regulation of secretion and to study this I needed methods for observing transport events in living cells with high spatial and time resolution. In this account, I shall present what I consider to be my key physiological and pathophysiological experiments on the exocrine pancreas, hoping that at least some of them will turn out to be of general relevance.

## Ion Channels Are Crucially Important for the Function of Exocrine Gland Cells

When I started research as an undergraduate medical student in Copenhagen in the late 1960s, histology and electron microscopy had already taught us much about the structure of the acinar cells. Furthermore, the pulse-chase experiments of George Palade and Jim Jamieson had, as already mentioned, revealed the crucial steps in the acinar secretory pathway.^1,10,11^ However, with regard to acute dynamic events, the pancreas—like all exocrine glands—was a “black box.” It was my particular aim to understand the intracellular mechanism by which secretion is acutely switched on when acinar cells are stimulated by ACh or CCK. At that time (late 1960s), this was “terra incognita.”

The acute activation of secretion was particularly striking in the case of my first study object, namely the cat submandibular gland. Cannulating the main duct from the gland and isolating the chorda tympani containing the parasympathetic nerve fibers to the gland, it was easy to observe the virtually instantaneous flow of saliva upon electrical nerve stimulation. Furthermore, the flow of saliva stopped immediately when the electrical stimulation was discontinued. I was intrigued by these acute events.

In the 1960s, the field of epithelial transport physiology was dominated by work on the kidney and the intestines, essentially informed by the pioneering work of Hans Ussing on the frog skin,^12^ which was seen as a useful general transport model. However, in the intestines, kidney, and frog skin, fluid and electrolyte transport occur continuously, in contrast to what happens in the exocrine glands. The only theory for control of exocrine secretion was based on acute Cl^−^ pump regulation and the involvement of ion channels was explicitly rejected.^13–15^ In general, acute regulation of ion channels was regarded as functionally important in nerve and muscle cells, but not in epithelia.

Lundberg’s transport model for salivary secretion was published in 1958 as part of a review article in *Physiological Reviews*.^15^ Lundberg’s key experimental evidence, indicating that the ACh-elicited hyperpolarization of the acinar cell membrane was fundamentally different from inhibitory post-synaptic potentials in the nervous system, had been published in *Nature* a few years earlier.^13^ Lundberg found that the ACh-elicited membrane potential change did not exhibit a reversal potential and was not associated with any change in membrane conductance. Since it was dependent on the presence of Cl^−^ in the external medium, he concluded that it must be due to direct activation of an electrogenic Cl^−^ pump.^14^ Lundberg was a well-known neuroscientist, who had worked, and published many papers, with the Nobel Laureate John Eccles,^16,17^ so his work on the salivary glands was naturally regarded as state of the art and dominated the chapter on secretion mechanisms in Arnold Burgen’s 1961 Monograph for the Physiological Society on “Physiology of the salivary glands.”^18^ It is therefore understandable that when I and a fellow undergraduate medical student published a paper in 1968, in the relatively obscure journal *Experientia*,^19^ contradicting the work of Lundberg, it had virtually no impact. We showed that the ACh-evoked hyperpolarization was undiminished when Cl^−^ in the perfusion fluid was replaced by sulphate,^19^ and we were right.

**Figure 2.**
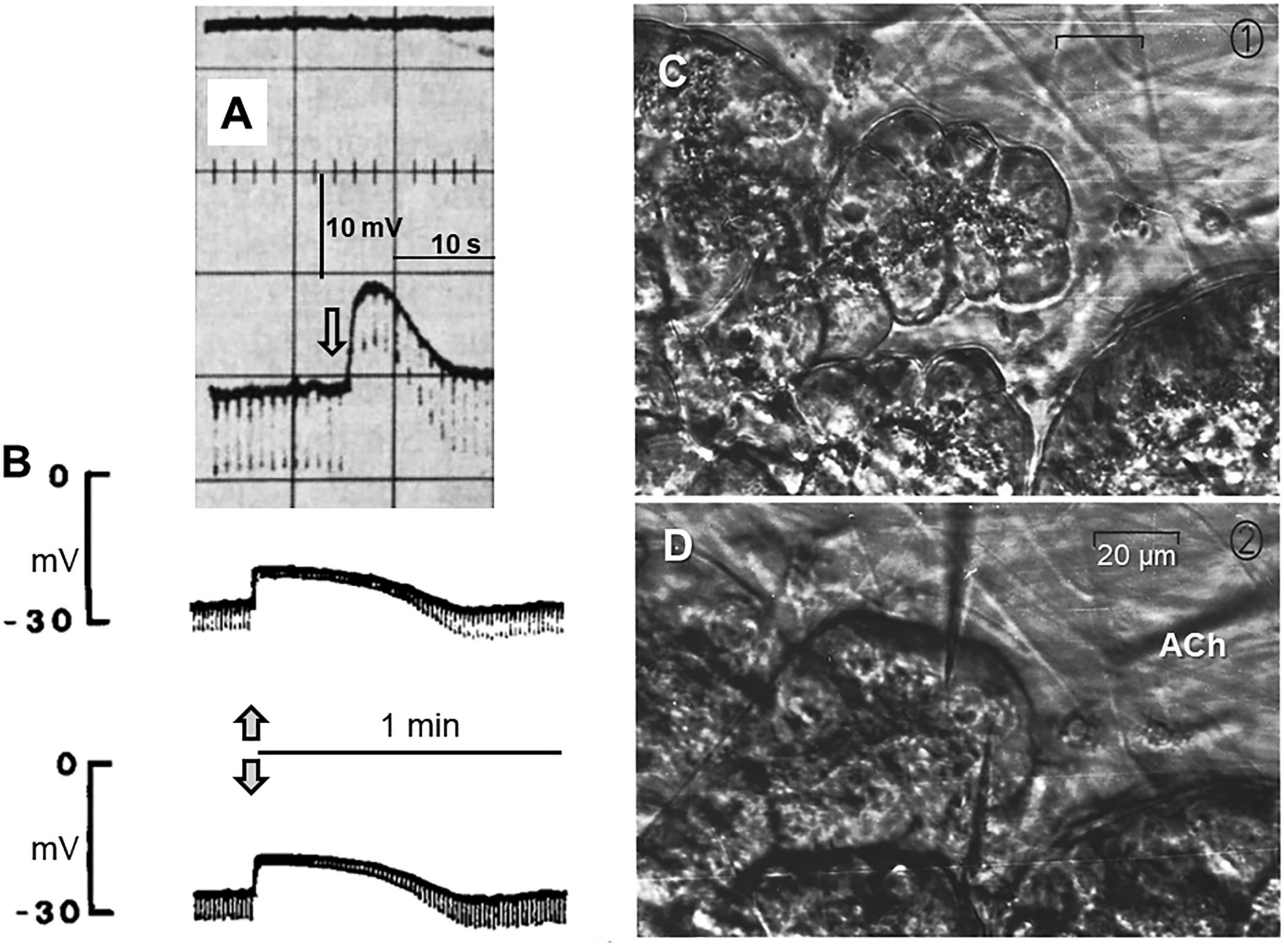
ACh elicits membrane depolarization and resistance reduction in pancreatic acinar cells. (A) A “blind” single microelectrode experiment on superfused segment of mouse pancreas. Arrow indicates ACh addition to the bath. Repetitive current pulses of constant magnitude were applied through the recording microelectrode (with tip resistance compensation) resulting in repetitive short-lasting hyperpolarizations. During the action of ACh the magnitude of the hyperpolarizations was reduced, indicating a reduction in membrane resistance, *i.e*., an increase in membrane conductance. (B) Experiment under direct visual control in which two separate microelectrodes were inserted into the neighboring acinar cells shown in C and D. ACh was applied through a third extracellular micropipette (seen in D) by ionophoresis. The arrows indicate the time of ACh application. Repetitive hyperpolarizing current pulses of constant magnitude were injected through the electrode from which the lower of the two recordings were made. As in A, the magnitude of the hyperpolarizations evoked by the injected current pulses are much diminished during the action of ACh, indicating a marked increase in membrane conductance. (C and D) Phase contrast microscopical pictures of the living cells in the acinar unit under investigation before (C) and after (D) insertion of the microelectrodes. A is adapted from Petersen 1973 ^22^ and B–D from Iwatsuki and Petersen 1978.^23^

The later finding, already mentioned, that ACh (and CCK) evoke depolarization, rather than hyperpolarization, of the pancreatic acinar cell membrane^2^ did not, of course, agree with Lundberg’s model. Furthermore, after my return to Copenhagen from the sabbatical in Cambridge, I was able to show that the ACh-elicited depolarization of the pancreatic acinar cell membrane was associated with a marked reduction in membrane resistance (Figure 2). It was now clear, at least to me, that ACh opened channels in the surface cell membrane. Soon after, Akinori Nishiyama from Tohoku University in Sendai, the first visiting research fellow to my laboratory in the Physiology Department at the University of Copenhagen, showed that also the ACh-evoked hyperpolarization of the acinar cell membrane in the submandibular gland was associated with a marked reduction in membrane resistance.^20^ When I had the opportunity to write my first major review article, which was published in *Physiological Reviews* in July 1976, soon after I had taken up the Symers Chair of Physiology at the University of Dundee in Scotland, I could confidently state that neurotransmitters and hormones evoke opening of ion channels in epithelial gland cells. Whether this results in depolarization or hyperpolarization or a mixture of both, depends on the balance between the conductance of pathways with different ion selectivity.^21^

With regard to monitoring acute cellular events, an electrophysiological approach had always been attractive to me because of the high time resolution that can be achieved. However, it was a severe limitation that we originally had to work “blind” by simply inserting a sharp microelectrode into a glandular tissue and record changes in membrane potential.^2,22,20^ It was impossible, using the dissecting microscopes at our disposal, to see the cells into which the microelectrode had been inserted. However, all histological work on the exocrine pancreas indicated that the vast majority of cells were acinar, and we therefore assumed that our recordings reflected events in these cells. The other important assumption was that ACh and CCK only acted on acinar cells. In retrospect, these assumptions seem quite bold and are, in fact, not 100% correct but, in practice, it turned out that they were “good enough” and the results from this early period of glandular investigations (up to 1977) are reproducible. It was nevertheless always one of my goals that we should be able to see the cells we were investigating. Our work had actually little impact until we were able to do this.

## “You Can Observe a Lot By Just Watching” (Yogi Berra): It Is Useful to Monitor Physiological Events in the Living Tissue in Real Time

Direct visualization of the acinar cells in the living pancreas only became possible after I had moved to the University of Dundee in 1975. There were of course many phase-contrast microscopes available, with sufficient magnification to allow inspection of individual cells, but I needed a microscope with a long working distance so that there would be adequate access for the microelectrodes to be inserted into the tissue. With the help of the Chief Technician in the Physiology Department (sadly, now an extinct species!), I found the solution from a completely different field. A phase-contrast mirror objective with an impressively long working distance (2 cm), that had originally been designed for the inspection of metal surfaces, turned out to be perfect for my purpose. Using this microscope, my first PhD student, Noriyuki Iwatsuki (a young surgeon from Tohoku University’s Medical School in Sendai) managed to record ACh-elicited membrane potential and resistance changes simultaneously from two neighboring acinar cells under direct visual control (Figure 2B–D). The success of this approach also relied on our choice of study object, namely the mouse pancreas. It is extremely thin, almost transparent, and at the edge of the isolated tissue, mounted on a perspex platform in a tissue bath, one could clearly see individual acinar cells (Figure 2C and D) without any need for microdissection or chemical treatment.

**Figure 3.**
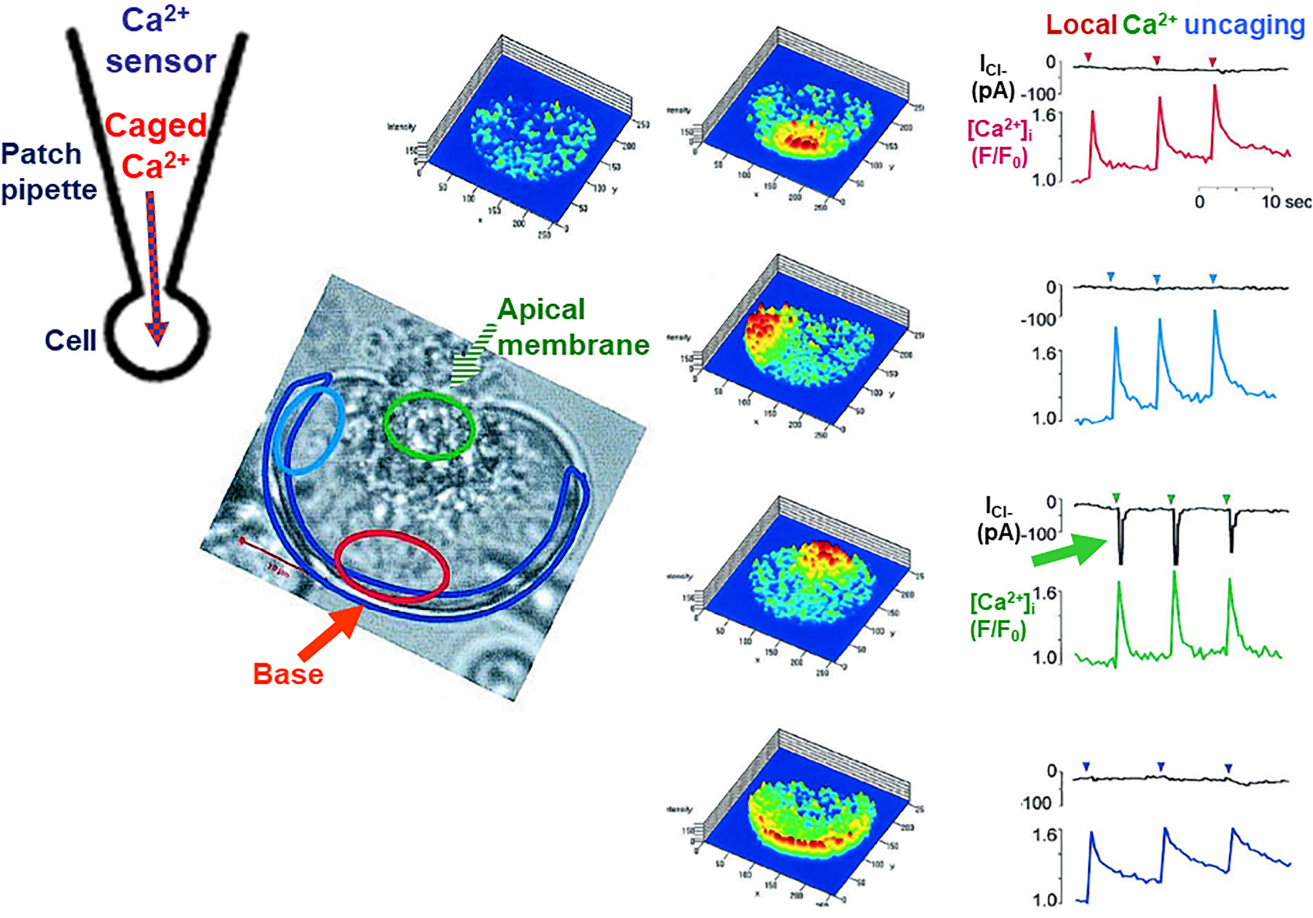
Ca^2+^ activation of Cl^−^ channels in the apical (luminal) membrane of an isolated pancreatic acinar cell. All recordings and images are from the same experiment on the same acinar cell. The patch pipette contained both the caged Ca^2+^ compound NP-EGTA and the fluorescent Ca^2+^ sensor Fluo-4. The transmitted light image shows the polarized nature of the isolated cell and the color-coded outlines indicate the specific subcellular locations that correspond to the [Ca^2+^]_i_ traces shown to the right that are associated with the fluorescence recordings demonstrating the localized Ca^2+^ signals generated by the uncaging of caged Ca^2+^ by UV light. It is only when Ca^2+^ is uncaged in the apical (green) region that the Cl^−^ current is switched on (green arrow). Adapted from Park et al. 2001.^25^

Noriyuki and I mapped the electrical communication network in the pancreas. It turned out that all acinar cells in an acinus were completely electrically coupled, whereas acinar cells from different acini were completely isolated from each other.^23^ Interestingly, we also showed that fully coupled cells within the same acinus could be completely and reversibly uncoupled from each other with supramaximal ACh stimulation.^23^ Most importantly, we were able to show that intracellular injection of Ca^2+^ could mimic the effect of external ACh application.^24^ This was the earliest evidence for Ca^2+^-mediated opening of Cl^−^ channels in the cell membrane. That these channels were selectively located in the apical cell membrane could only be proven many years later, when I was at the University of Liverpool. This, again, depended on direct visual inspection of the acinar cell under investigation. These difficult experiments^25^ (Figure 3) were carried out by Myoung Kyu Park from Seoul National University, who was a visiting research fellow in my laboratory. His work required the combination of patch clamp electrophysiology to record the Ca^2+^-activated Cl^−^ current with imaging at subcellular resolution of the distribution of [Ca^2+^]_i_ and the ability to uncage caged Ca^2+^ at specific preselected regions of the cell (Figure 3).

The invention of the gigaseal high-resolution method for recording single channel and whole cell currents^26^ had an enormous impact on the field of electrophysiology. In a recent autobiographical article,^27^ I have described my participation in the early days of this revolutionary technique and how my laboratory, very shortly after I had taken up the George Holt Chair of Physiology at the University of Liverpool in England, became the first to publish single channel^28^ and whole cell^29^ current recordings from epithelial cells. By comparing single channel and whole cell current recording of high-conductance Ca^2+^- and voltage-activated K^+^ channels, we were also able, for the first time, to quantify the number (∼50) of such channels per cell.^29^ These early papers, summarized in a review article in *Nature* published in 1984,^30^ proved in a way that was beyond dispute, that epithelial cells did have several types of ion channels with characteristics similar to those found in nerve and muscle cells. It is, however, important to stress that they do not possess voltage-activated Ca^2+^ channels.^31^ These studies also showed that the most important ion channels in exocrine gland cells were controlled by receptor-activated changes in [Ca^2+^]_i_.^28,29,32,33^ The work in this period resulted in the formulation of the first coherent model of acinar fluid secretion, focusing on Ca^2+^-activation of Cl^−^ channels in the apical membrane and K^+^ channels in the basolateral membrane.^34^ I reviewed these data in detail, when I gave the Physiological Society’s Annual Review Prize Lecture in 1991, which was subsequently published in *J Physiol*.^31^

## Physiological Ca^2+^ Signals

**Figure 4.**
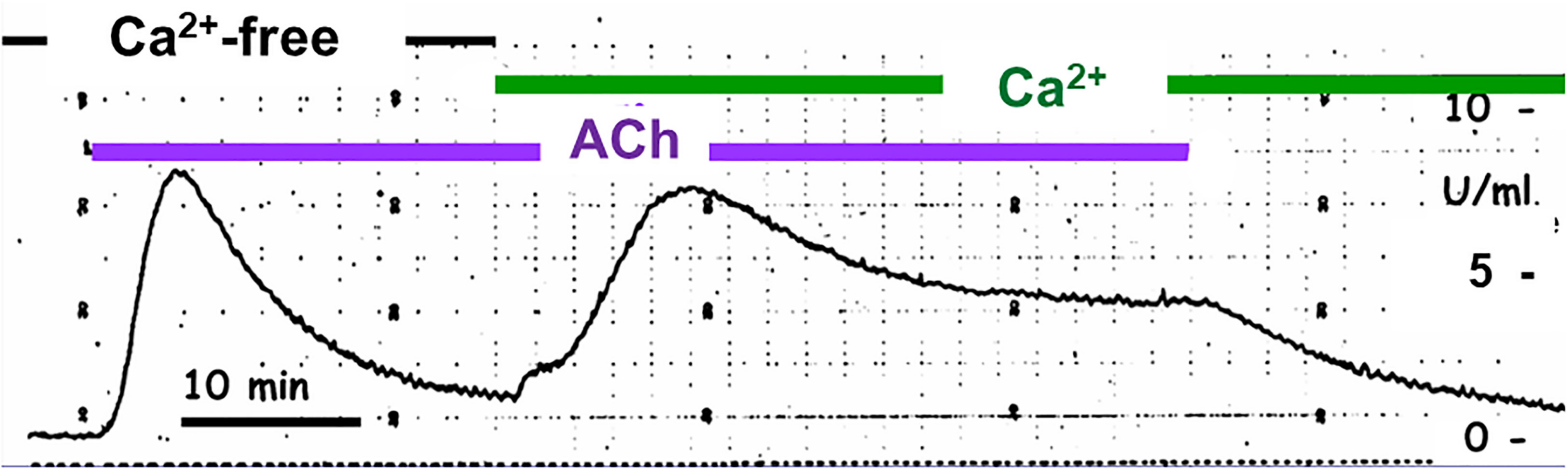
Continuous recording of amylase output from superfused segments of mouse pancreas. ACh (1 µm) evokes a marked increase in amylase output in the absence of Ca^2+^ in the superfusion fluid, but the effect is only transient. Addition of Ca^2+^ to achieve an extracellular [Ca^2+^]_i_ of 2.6 mM promotes a second marked rise in amylase output leading to a sustained secretion, which stops when the ACh stimulation is discontinued. Adapted from Petersen and Ueda 1976.^38^

As already mentioned in the first section of this article, the importance of Ca^2+^ for secretion, also in the exocrine glands, was already recognized in the early 1970s.^2,3,35,36^ Unfortunately, most studies of the exocrine glands were rather crude and had poor time resolution. As an electrophysiologist, I was always attracted to experiments in which events could be recorded continuously as they happened and during my period in Cambridge in the early 1970s, I was part of a team that developed an automated fluorescence method for measurement of amylase output from superfused small fragments of pancreas.^37^ With this technique, one can monitor continuously, and with relatively good time resolution, the enzyme secretion from the pancreas^38^ and observe directly the effect of, for example, stimulation with ACh (Figure 4). One of the most important results of the use of this technology was the very clear separation of two phases of stimulant-elicited secretion, an initial phase that did not require external Ca^2+^ and a sustained phase that was acutely dependent on extracellular Ca^2+^ (Figure. 4).

**Figure 5.**
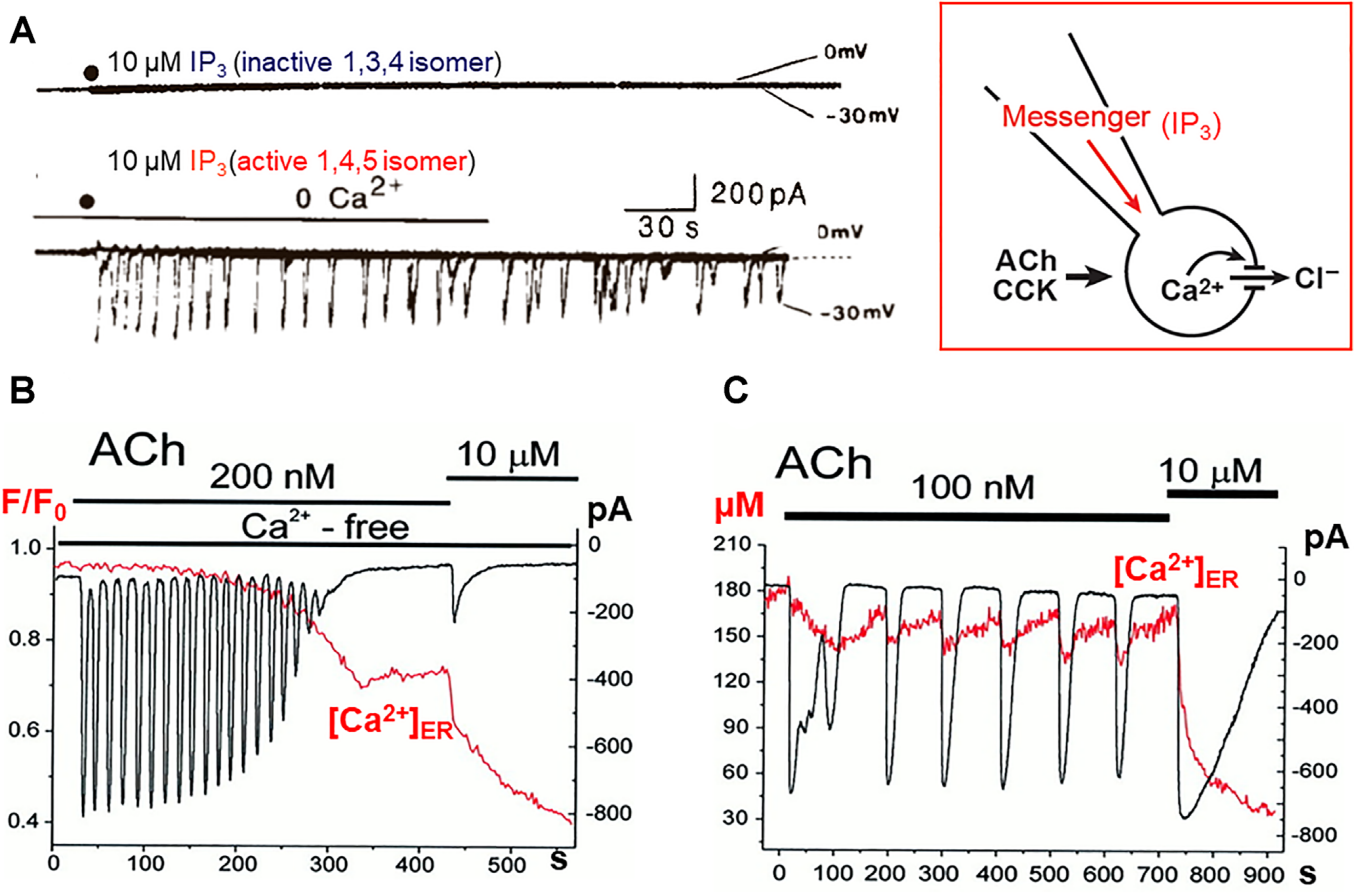
IP_3_ and ACh evoke repetitive spikes of Ca^2+^-activated Cl^−^ currents in isolated pancreatic acinar cells. (A) Two separate patch clamp whole cell current recording experiments with the inactive 1,3,4-isomer and active 1,4,5-isomer of IP_3_, respectively. The Cl^−^ concentration in the extracellular solution and the pipette solution were identical, so there is no electrochemical Cl^−^ gradient when the membrane potential is held at 0 mV, but at −30 mV clear inward current spikes are evoked by the active IP_3_. (B and C) Combined recording of Ca^2+^-activated Cl^−^ current (black trace) and [Ca^2+^]_ER_ (red trace). In B, changes in [Ca^2+^]_ER_ were monitored with the help of Fluo-4, whereas in C MagFura-2 was used. A is adapted from Wakui et al. 1989 ^42^ and B and C from Park et al. 2000.^43^

With regard to the salivary glands, I had already in 1972 provided evidence, based on radioactive Ca^2+^ tracer studies, for ACh-elicited release of Ca^2+^ from internal stores^35^ and this had been confirmed also for the pancreas the following year.^3,36^ The problem of how a signal (for example, ACh) acting on the outside of a cell could cause opening of Ca^2+^ release channels in the ER had been solved in 1983 by the experiments of Irene Schulz and Michael Berridge on permeabilized pancreatic acinar cells, in which inositol 1,4,5-trisphosphate (IP_3_) had been shown to release Ca^2+^ from a nonmitochondrial store.^39^ IP_3_ very quickly became recognized as the ubiquitous intracellular messenger releasing Ca^2+^ from internal stores.^40^ Employing the Ca^2+^-activated Cl^−^ channel in the apical cell membrane as an endogenous Ca^2+^ sensor (Figure 3), Makoto Wakui and I showed that the active 1,4,5 isomer of IP_3_ elicited repetitive Ca^2+^ spikes. This spiking could continue for several minutes in the complete absence of external Ca^2+^ (Figure 5A) and this was also the case with the Ca^2+^ spikes elicited by ACh (Figure 5B). The spikes evoked by ACh were blocked when the IP_3_ receptor antagonist heparin was included in the intracellular pipette solution as were those elicited by IP_3_.^41^

It had become clear already in the early 1970s that the pancreatic acinar cells were electrically nonexcitable, because depolarizing the cell membranes by exposure of the pancreatic tissue to a solution with a high concentration of K^+^ did not elicit Ca^2+^ movements and secretion,^3,44^ as it did in endocrine cells.^45^ In these experiments, on the pancreas from the mouse and the cat, it was necessary to use atropine to block the muscarinic receptors on the acinar cells, as otherwise the K^+^ depolarization of the parasympathetic nerve endings around the acini would release ACh, which would then activate intracellular Ca^2+^ release and secretion.^3^ Nevertheless, George Scheele—a member of Palade’s research group—published papers in 1978 and 1980 indicating that in the guinea pig pancreas, K^+^ depolarization in the presence of atropine elicited exocytotic enzyme secretion and that this secretory response was acutely dependent on the presence of Ca^2+^ in the external solution.^8,9^ This result seemed to vindicate Palade’s original idea that pancreatic secretion, like secretion of neurotransmitters from nerve endings or hormones from endocrine glands, was initiated by membrane depolarization opening voltage-dependent Ca^2+^ channels. I suspected that there was a simpler explanation for the discrepancy between these results^8,9^ and those obtained by Barry Argent^44^ and myself.^3^ Given that cyclic AMP is a strong stimulator of secretion from the parotid gland,^7^ it would seem possible that in the guinea pig pancreas there would be release from nerve endings not only of ACh, but also a co-transmitter. Such a co-transmitter might activate receptors on the acinar cells linked to adenylate cyclase resulting in cyclic AMP generation. So, my collaborators and I set out to test this hypothesis. We reproduced the basic result that Scheele had published, namely that in the guinea pig pancreas K^+^ depolarization, as well as direct electrical stimulation of intrinsic nerves in the tissue, evoked enzyme secretion that could not be blocked by atropine. The secretory response was reduced by atropine application but was still substantial.^46,47^ The enzyme secretion was not associated with any measurable Ca^2+^ movement,^46^ but was associated with a clear increase in cyclic AMP concentration and could be mimicked by application of vaso-active intestinal polypeptide (VIP).^47^ We therefore concluded that the guinea pig pancreatic acinar cell was electrically nonexcitable, exactly like the mouse pancreatic acinar cell, but possessed VIP receptors activating cyclic AMP production.^46,47^ More recently, we have been able to observe neuro-glandular transmission in the mouse pancreas, by measuring directly and simultaneously [Ca^2+^]_i_ in a nerve ending and its adjacent acinar cell.^48^ K^+^ depolarization evoked a Ca^2+^ signal first in the nerve cell and then in the acinar cell. However, when atropine was applied, K^+^ depolarization only evoked a Ca^2+^ signal in the nerve cell.^48^

When it became possible to combine electrophysiological recordings with measurement of [Ca^2+^] inside the ER ([Ca^2+^]_ER_), it could be seen that the predominant signaling pattern of repetitive short-lasting spikes could continue for a few minutes in the absence of external Ca^2+^, without any observable reduction of [Ca^2+^]_ER_. However, when [Ca^2+^]_ER_ began to decline, the amplitude of the Ca^2+^ spikes decreased sharply and finally spiking ceased at a point when the ER was far from empty of Ca^2+^ (Figure 5B). It was only when ACh occasionally elicited broader Ca^2+^ spikes that it was possible to observe a concomitant sharp reduction in [Ca^2+^]_ER_ and a subsequent slower Ca^2+^ reuptake in the inter-spike period (Figure 5C).

**Figure 6.**
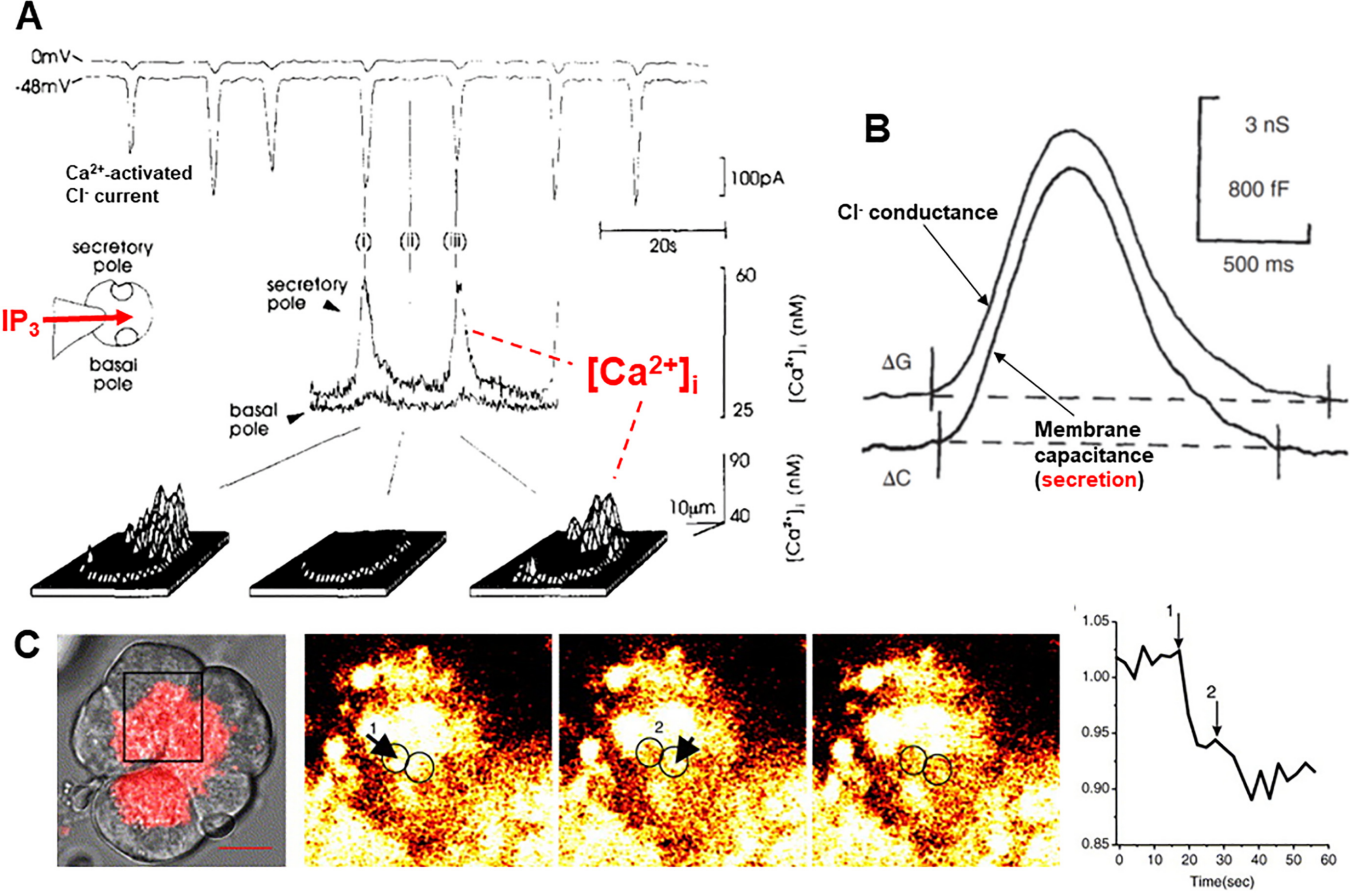
IP_3_ and ACh evoke repetitive short-lasting [Ca^2+^]_i_ spikes localized in the apical granule-rich region and these evoke exocytotic secretion. (A) Patch clamp recording of Ca^2+^-activated Cl^−^ current combined with imaging of [Ca^2+^]_i_ changes. Local (apical) [Ca^2+^]_i_ spikes are seen to occur synchronously with the Cl^−^ current spikes. (B) A single Ca^2+^ spike (from a train of repetitive IP_3_-elicited spikes) is monitored by recording the Cl^−^ conductance change simultaneously with the change in capacitance. (C) Stepwise reduction in localized quinacrine fluorescence during ACh stimulation in an isolated acinar unit. A is adapted from Thorn et al. 1993,^49^ B from Maruyama and Petersen 1994, ^51^ and C from Park et al. 2004.^52^

Imaging revealed that the Ca^2+^ spikes occurred specifically in the apical granule-rich region (Figure 6A). Since, in these experiments, the whole of the cell interior was flooded with IP_3_ (or even with the nonmetabolizable IP_3_S), we concluded that the IP_3_ receptors on the ER must be concentrated in the apical region.^49^ When I presented these data at a seminar held at Tokyo University in March 1993, it transpired that Haruo Kasai from that institution had very similar data and had reached the same conclusion. Both of us had submitted papers reporting this finding to *Science* and both our papers had been rejected. At the time we met, we had both submitted our papers to *Cell* and were both battling with the editor to get them accepted. We were finally successful, and our papers were published in *Cell*, back to back, in August 1993.^49,50^

In order to test whether the short-lasting Ca^2+^ spikes were capable of triggering exocytotic enzyme secretion, we needed to employ a method for monitoring this process at high time resolution. This called for an electrophysiological approach. Neher and Marty had developed such a method, namely measurement of capacitance.^53^ The capacitance of cellular membranes is a biological constant and any insertion of membrane, as occurs in exocytosis, will increase the capacitance and the subsequent endocytic retrieval of the membrane will reduce the capacitance. By simultaneously recording membrane conductance and capacitance, it was possible to resolve the time course of the secretory process during an individual Ca^2+^ spike. As seen in Figure 6B, the two parameters essentially changed in parallel, although the rise in [Ca^2+^]_i_ (monitored by the Cl^−^ conductance) slightly preceded and slightly exceeded the capacitance rise, indicating that the Cl^−^ channels are a bit more sensitive to a rise in [Ca^2+^]_i_ than the exocytotic machinery. We also attempted to visualize the secretion process by monitoring the disappearance of the fluorescent agent quinacrine, which accumulates in acid compartments such as the ZGs. As seen in Figure 6C, sudden sharp reductions in fluorescence intensity could be seen during ACh stimulation, representing loss of granule content during exocytotic secretion.

There is no immediate opening of Ca^2+^ channels in the acinar cell membrane upon stimulation with either ACh or CCK.^3,35^ This explains the experimental observation that the initial part of the secretory response to stimulation is completely independent of the presence of Ca^2+^ in the external solution (Figure 4). However, during prolonged stimulation, secretion becomes acutely dependent on external Ca^2+^. This suggests that following the initial release of Ca^2+^ from the ER, there is Ca^2+^ inflow across the acinar cell membrane, which is needed to sustain secretion. This was first shown by Kondo and Schulz in 1976,^54^ but at that time the nature of the influx pathway was unknown. In 1992, Hoth and Penner identified and characterized an inward Ca^2+^ current in mast cells elicited by depleting the ER of Ca^2+^.^55^ This inward Ca^2+^ flow is known as the Ca^2+^ Release Activated Ca^2+^ (CRAC) current and has been studied extensively by Anant Parekh and others, particularly in immune cells.^56^ The CRAC channels are extremely Ca^2+^ selective and have a very low single channel conductance, making it impossible to record single channel currents.^56^ Even the maximally activated whole cell CRAC current, elicited by complete emptying of the ER Ca^2+^ store, is typically only a few pA ^55^ and therefore difficult to record in many cell types.

**Figure 7.**
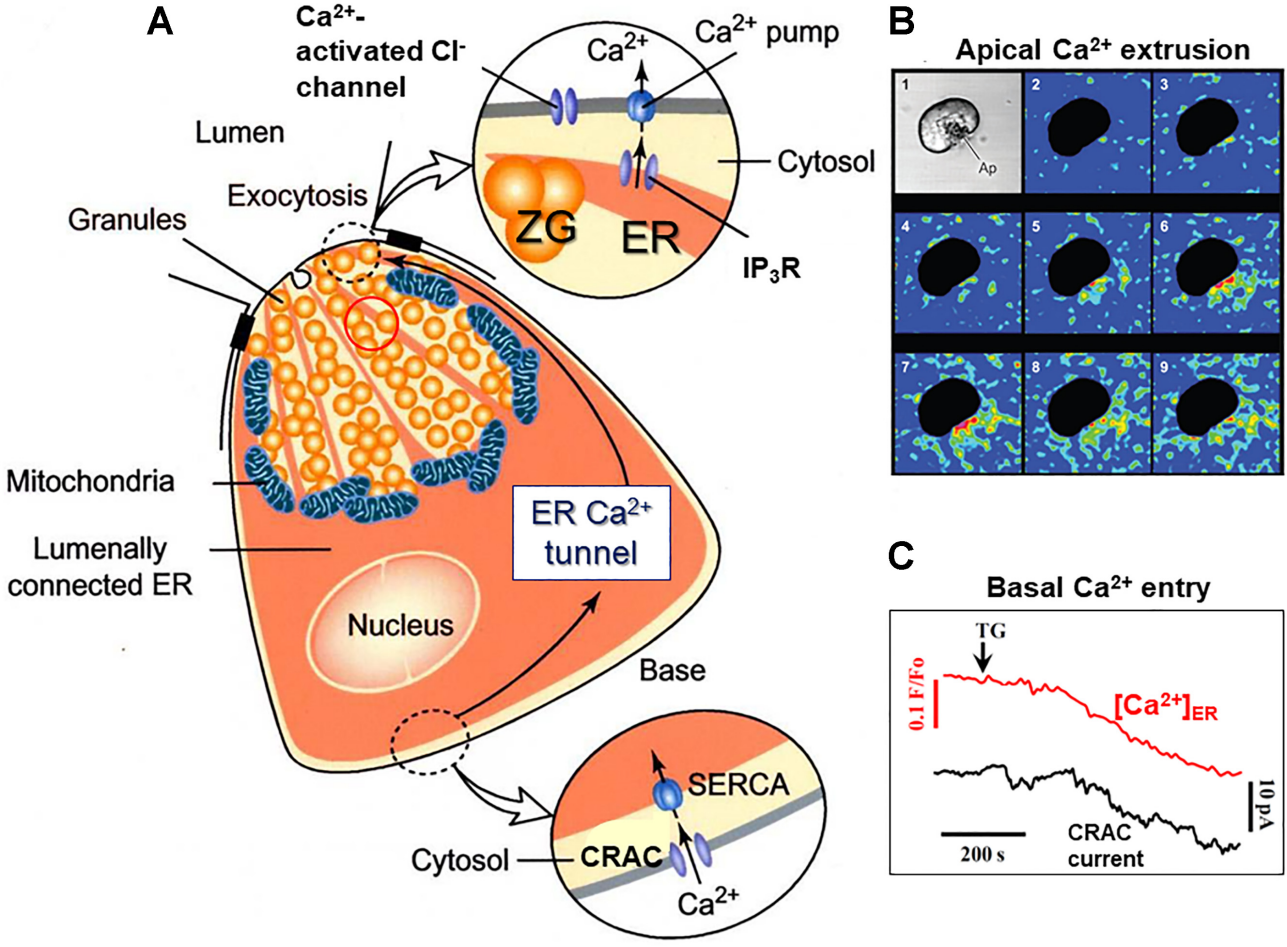
Ca^2+^ exit at the apical pole and compensatory Ca^2+^ entry at the base of acinar cells. (A) Schematic diagram illustrating the overall Ca^2+^ transport events during stimulation with either ACh or CCK. At the apical (upper) pole, exocytosis, and opening of Cl^−^ channels have been activated by the local high [Ca^2+^]_i_, which has also activated the plasma membrane Ca^2+^ pump. At the base, CRAC channels have been opened as a consequence of the reduction in [Ca^2+^]_ER_ and Ca^2+^ is flowing in through these channels and immediately taken up into the ER by Ca^2+^ pumps (SERCA—Sarco-ER Ca^2+^ activated ATPase). Ca^2+^ can diffuse easily in the lumen of the ER and thereby replenish Ca^2+^ lost from the apical ER terminals, where the IP_3_ receptors are localized (ER Ca^2+^ tunnel function). (B) ACh-elicited extrusion of Ca^2+^ specifically across the apical membrane. An isolated pancreatic acinar cell is placed in a solution containing the fluorescent Ca^2+^ sensor Calcium Green-1 bound to high molecular weight (500 000) dextran, limiting the diffusion of the Ca^2+^ sensor. The optical section goes through the cell, which is made black. The images are taken at 3 s intervals and ACh is added between images 2 and 3, The changes in color from blue, green to yellow, and red represent rises in the extracellular [Ca^2+^]_i_. (C) Combined recording of [Ca^2+^]_ER_ and inward Ca^2+^ current following application of thapsigargin (TG). TG arrests the SERCA pump and it is seen that as [Ca^2+^]_ER_ gradually decreases the inward Ca^2+^ current increases. A is adapted from Petersen et al. 2001,^57^ B from Belan et al. 1996, ^58^ and C from Gerasimenko et al. 2013.^59^

We only succeeded in recording the CRAC current in the pancreatic acinar cells after I had moved to Cardiff University in 2010 to become Director of the School of Biosciences, succeeding the Nobel Laureate Martin Evans in this post. These experiments were carried out by Oleksiy Gryshchenko from the Bogomoletz Institute of Physiology in Kyiv, who became a regular visiting senior research fellow. He was also able to monitor the development of the current after poisoning the ER Ca^2+^ pumps with thapsigargin, at the same time as he recorded the loss of Ca^2+^ from the ER (Figure 7).

The cardinal features of Ca^2+^ handling in the polarized acinar cell are illustrated in Figure 7. The primary ACh-elicited Ca^2+^ release occurs in the apical region where the ZGs are concentrated and the rise in the local [Ca^2+^]_i_ activates both exocytosis and the Cl^−^ channels in the apical membrane. Although much of the Ca^2+^ released during a single Ca^2+^ spike is taken up again into the apical ER via the ER Ca^2+^ pumps,^60^ some of the Ca^2+^ is lost across the apical membrane due to activation of the plasma membrane Ca^2+^ pumps that are predominantly located in the apical membrane (Figure 7B). Therefore, in the absence of external Ca^2+^, spiking gradually diminishes and finally stops (Figure 5), but in the normal physiological situation, the lost Ca^2+^ is replenished via the CRAC channels in the basolateral membrane (Figure 7A and C).

The function of the CRAC channels in the acinar cells is to refill the ER with Ca^2+^ after the inevitable loss that occurs during Ca^2+^ spiking. We know that this refilling occurs across the basal membrane, because the whole of the ER can be reloaded with Ca^2+^ via a single cell-attached Ca^2+^-containing pipette at the base of the cell.^61^ The overall function of this system depends on the whole of the ER having one continuous lumen through which Ca^2+^ can diffuse easily. We have shown this by direct observation of Ca^2+^ movements in the ER lumen.^43^ In fact, Ca^2+^ diffuses much more easily in the lumen of the ER than in the cytosol, because the calcium binding capacity in the cytosol is much higher than in the ER lumen.^57^ Ca^2+^ lost from the apical ER elements during ACh-elicited Ca^2+^ spiking can therefore easily be replenished from the much larger Ca^2+^ store at the base, which in turn can be reloaded by Ca^2+^ entry through the basal CRAC channels (Figure 7).

## The Special Location and Function of the Mitochondria

**Figure 8.**
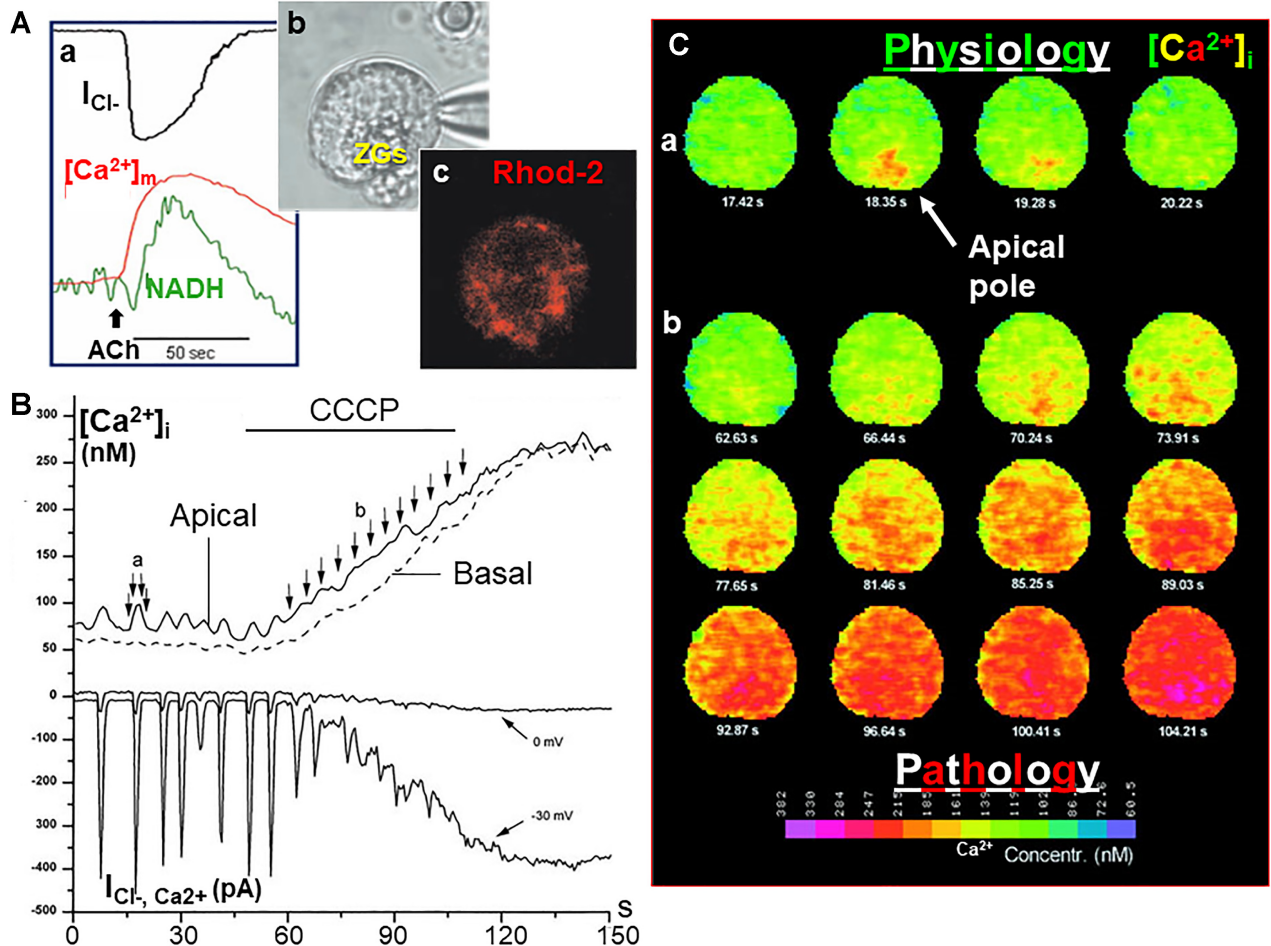
Stimulus-metabolism coupling and the shaping of cytosolic Ca^2+^ signals by functioning mitochondria. (Aa) Triple recording of Ca^2+^-activated Cl^−^ current, change in the mitochondrial [Ca^2+^] ([Ca^2+^]_m_) and NADH autofluorescence, following ACh stimulation. (Ab) Transmitted light picture of the acinar cell under investigation. (Ac) The peri-granular mitochondrial belt highlighted by increased Ca^2+^-sensitive Rhod-2 fluorescence. The positively charged Rhod-2 accumulates inside the mitochondria due to the very large electrical potential across the inner mitochondrial membrane. (B and C) Combined patch clamp whole cell current recording of Ca^2+^-sensitive Cl^−^ current and imaging of [Ca^2+^]_i_ changes during intracellular IP_3_ infusion. The repetitive local Ca^2+^ spikes elicited by IP_3_ are gradually transformed into a sustained global [Ca^2+^]_i_ elevation following application of the ionophore CCCP, depolarizing the inner mitochondrial membrane. A is adapted from Voronina et al. 2002.^63^ B and C are adapted from Tinel et al. 1999.^62^

In contrast to the ease with which Ca^2+^ diffuses in the ER lumen, Ca^2+^ movement in the cytosol is severely restricted. The most important obstacle to Ca^2+^ spikes being transmitted from the apical granule-rich region to the base of the cell is the special distribution of the mitochondria. We discovered this by an unexpected but, as it turned out, important observation made in response to a question from a reviewer about the localization of mitochondria in pancreatic acinar cells. I asked Hana Tinel (a visiting research fellow from the Max Planck Institute for Molecular Physiology in Dortmund) to look for mitochondria in living pancreatic acinar cells.^62^ Many EM pictures of acinar cells had been published in numerous articles that had not shown any particular distribution of the mitochondria, so it was a great surprise for us to see that in the living acinar cells there was a very distinct distribution of these organelles. By far the highest density of mitochondria was seen surrounding the apical granular region. The striking appearance of what we termed a mitochondrial belt (Figures 7 and 8), separating the granular region from the basolateral part of the acinar cell, surrounding the nucleus, suggested an interesting physiological role and a specific hypothesis.

Given that we had shown that physiological Ca^2+^ signaling occurs as repetitive short-lasting Ca^2+^ spikes confined to the apical granular region (Figure 6), we wanted to test whether this was dependent on functioning mitochondria. We showed that inhibition of mitochondrial function transformed IP_3_-mediated repetitive apical Ca^2+^ spikes into a sustained global [Ca^2+^]_i_ elevation (Figure 8B and C). These results indicated that under physiological conditions the peri-granular mitochondria stop Ca^2+^ waves progressing from the apical pole toward the base by taking up Ca^2+^ quickly and only release it much more slowly. We subsequently showed, by direct measurements of [Ca^2+^] changes in the mitochondria, that this was indeed the case (Figure 8A). We also showed that the [Ca^2+^] rise inside the mitochondria was driving the dehydrogenases in the Krebs cycle, as seen by a rise in the concentration of NADH (Figure 8A).

## Toxic Ca^2+^ Signals

Noting that a prolonged excessive rise in [Ca^2+^]_i_ causes cell death in many different tissues, Robert Sutton and I proposed in 1995 that toxic Ca^2+^ signals could be the trigger for acute pancreatitis (AP).^64^ Having identified repetitive local Ca^2+^ spikes in the apical region as the physiological regulators of pancreatic enzyme secretion,^49,51^ later also shown in human acinar cells,^65^ the hypothesis that a sustained global [Ca^2+^]_i_ elevation might be toxic seemed plausible.^49^

The experiment shown in Figure 8, in which mitochondrial depolarization transforms local Ca^2+^ signals to a global sustained [Ca^2+^] elevation, became the starting point for our subsequent focus on the role of mitochondrial inhibition in the development of AP. It would gradually become clear that excessive release of Ca^2+^ from internal stores, causing pathological opening of CRAC channels and therefore excessive Ca^2+^ entry, would overwhelm the mitochondria, and open a large channel in the inner mitochondrial membrane (the mitochondrial permeability transition pore—MPTP). The opening of this pore caused mitochondrial depolarization^66^ and thereby stopped ATP generation.^67^ This further exacerbated the toxic effect of the prolonged [Ca^2+^]_i_ rise, by disabling the ATP-dependent Ca^2+^ pumps that normally extrude excess Ca^2+^ from the cytosol. It became abundantly clear that inhibition of mitochondrial function is a crucial step in the process of acinar necrosis that is such a critical element in the development of AP.^66^ It follows from these findings that preventing the opening of the MPTP should protect against the development of AP. This prediction has turned out to be correct. When MPTP opening was blocked pharmacologically or genetically, all the cellular changes associated with AP were abolished or severely reduced in four different models of experimental AP.^68^

## Intracellular Trypsinogen Activation

I was impressed by the early studies of Babkin,^69^ further developed many years later by Lampel and Kern,^70^ demonstrating vacuole formation inside acinar cells following hyperstimulation. Could this be related to toxic Ca^2+^ signals? We found that stimulation with nanomolar, rather than the physiological low picomolar, CCK concentrations, evoked a sustained global [Ca^2+^]_i_ elevation and induced vacuole formation.^71^ Importantly, this vacuole formation could be prevented by loading the cells with a Ca^2+^ chelator or simply by omitting Ca^2+^ from the external solution.^71^ We also utilized a probe that becomes fluorescent after trypsin cleavage of two oligopeptide side chains and could in this way visualize, in the living tissue, the increase in intracellular trypsin concentration elicited by CCK hyperstimulation. Our live imaging of the subcellular distribution of trypsin activity showed that trypsin activity appeared initially in the apical granular area,^71^ as also reported at around the same time by Markus Lerch and his collaborators.^72^ However, the image resolution in both our study^71^ and the one from Lerch’s group^72^ was insufficient to determine the precise organellar localization.

The publication of our study^71^ was not without problems, as is often the case, particularly with important new data. We first submitted the paper to *Gastroenterology* but, after several rounds of reviewing and revision, I had the feeling that we were not going to be able to persuade the journal to accept our study, so we decided to send the manuscript to *PNAS*. At that time, this was done via a member of the National Academy who, by mistake, used surface rather than air mail to send our paper from Cambridge in England to the Washington office of *PNAS*. It therefore took a couple of months before the paper arrived in the US. Happily, our bad luck then finally ended, and the paper was not only accepted, but also highlighted by an excellent commentary written by Anant Parekh.^73^

**Figure 9.**
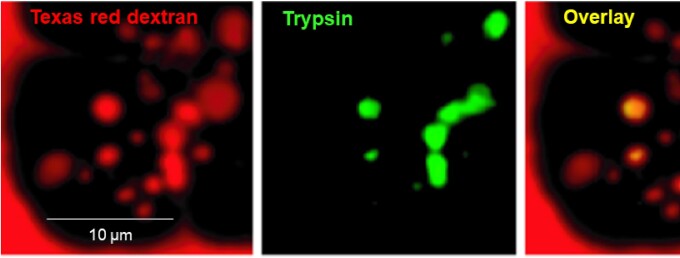
Trypsinogen activation in post-exocytotic endocytic vacuoles following CCK hyperstimulation (10 nM CCK) for 2 h and 30 min. The small acinar cell cluster had been placed in a solution containing Texas red dextran and the trypsin-sensitive fluorescent probe BZiPAR. Trypsin activity is seen co-localized with Texas red dextran inside the acinar cells. Adapted from Sherwood et al. 2007.^74^

In order to progress, we formulated a very specific hypothesis, namely that the vacuoles were post-exocytotic endocytic structures and that the initial trypsinogen activation occurred in the resulting endosomes (vacuoles). To test this, it was essential to get the trypsin-sensitive probe into the endocytic compartment and this was accomplished by including the probe in the external bathing solution. Isolated acinar cells were then stimulated by a high (nanomolar) CCK concentration. As seen in Figure 9, trypsin activity was clearly detected in vacuolar structures that were endocytic, because they also contained the membrane-impermeant macromolecule Texas red dextran which had been placed in the extracellular solution. This agent could only have been taken up into the cell by endocytosis.^74^

The biophysical mechanism underlying trypsinogen activation is still unclear. The major part of the content of the ZG is not in free solution but bound in the granular matrix, held together by Ca^2+^. The matrix may behave as an ion exchanger and if Ca^2+^ could be replaced by a mono-valent cation, it would disaggregate. This could happen after fusion of the ZG with the apical cell membrane and opening at the point of fusion, because the granule interior would then be in direct contact with the fluid in the acinar lumen, which has a high Na^+^ concentration.^75^ Ion exchange could be part of the normal physiological secretion process, as the pro-enzymes need to be free in order to become working enzymes in the gut. However, in the pathological state of hyperstimulation, the excessively high [Ca^2+^]_i_ in the apical pole may provide an extra strong stimulus for endocytosis, so that the enzymes rather than being secreted, which would require fusion pore expansion,^76^ would be taken back into the cell. There is, to the best of my knowledge, no evidence for this hypothesis, but it is at least a plausible way of thinking about the process of intracellular trypsinogen activation.

## There Is a Need for Realistic Disease Models

One valid criticism of both our studies,^71,74^ as well as those from the groups of Saluja^77^ and Lerch,^72^ was the exclusive use of CCK hyper-stimulation to induce AP-like changes in the acinar cells. AP in real life is not happening as a result of high (nanomolar) CCK concentrations in the blood, but mostly as a result of gallstone complications or alcohol abuse. CCK hyperstimulation could activate several G-proteins and may therefore result in intracellular actions that could be different from those elicited by the pathophysiologically more relevant actions of alcohol or bile acids. In later studies, we therefore discontinued the CCK hyperstimulation protocol and instead focused on ethanol.

I had anticipated that exposure of isolated pancreatic acinar cells to high concentrations of ethanol would be destructive but, when we first tested the effect of ethanol in 2003,^78^ it turned out to be rather minor. Ethanol, even at the extremely high concentration of 850 mM, a much higher level than can be observed in blood after even the most extreme alcohol consumption,^79^ mostly only elicited a small elevation of [Ca^2+^]_i_. Remarkably, such a high ethanol concentration did not even prevent the acinar cells from regulating [Ca^2+^]_i_ back to the resting level after a supramaximal ACh stimulus had caused a major rise. This showed that the ATP-dependent Ca^2+^ pumps were fully functional in the presence of a very high alcohol concentration.^78^

We then found an important paper published in *Science* in 1988 by Laposata and Lange,^80^ indicating that the toxic effects of ethanol were mainly due to the action of nonoxidative ethanol metabolites rather than ethanol itself or its oxidative metabolite acetaldehyde. A simple hypothesis now suggested itself, namely that the prolonged and excessive [Ca^2+^]_i_ elevations that were destroying the pancreatic cells in AP were, in the case of alcohol-related AP, caused by the product of ethanol and fatty acids. Fatty acids can combine with ethanol to yield fatty acid ethyl esters (FAEEs) and this process is particularly effective in the exocrine pancreatic cells.^80^ We thus began to test the actions of palmitoleic acid (POA) and palmitoleic acid ethyl ester (POAEE) and quickly established that POAEE was a powerful releaser of Ca^2+^ from internal stores, eliciting a substantial and sustained elevation of [Ca^2+^]_i_.^66,78^ The mechanism by which POAEE releases Ca^2+^ from the ER, and also from acid stores^81^ is still unknown, but depends on functional IP_3_ receptors, as shown by knock-out experiments.^81^ The action of POAEE could be mimicked by many other FAEEs^78^ but, for practical reasons, we concentrated our efforts on studying the effects of POAEE. In contrast, acetaldehyde did not cause any Ca^2+^ signals and did not destroy the acinar cells.^78^ We have not studied the effects of bile acids as intensively as those of alcohol and fatty acids, but they are rather similar to what has been observed with POAEE.^82,83^

**Figure 10.**
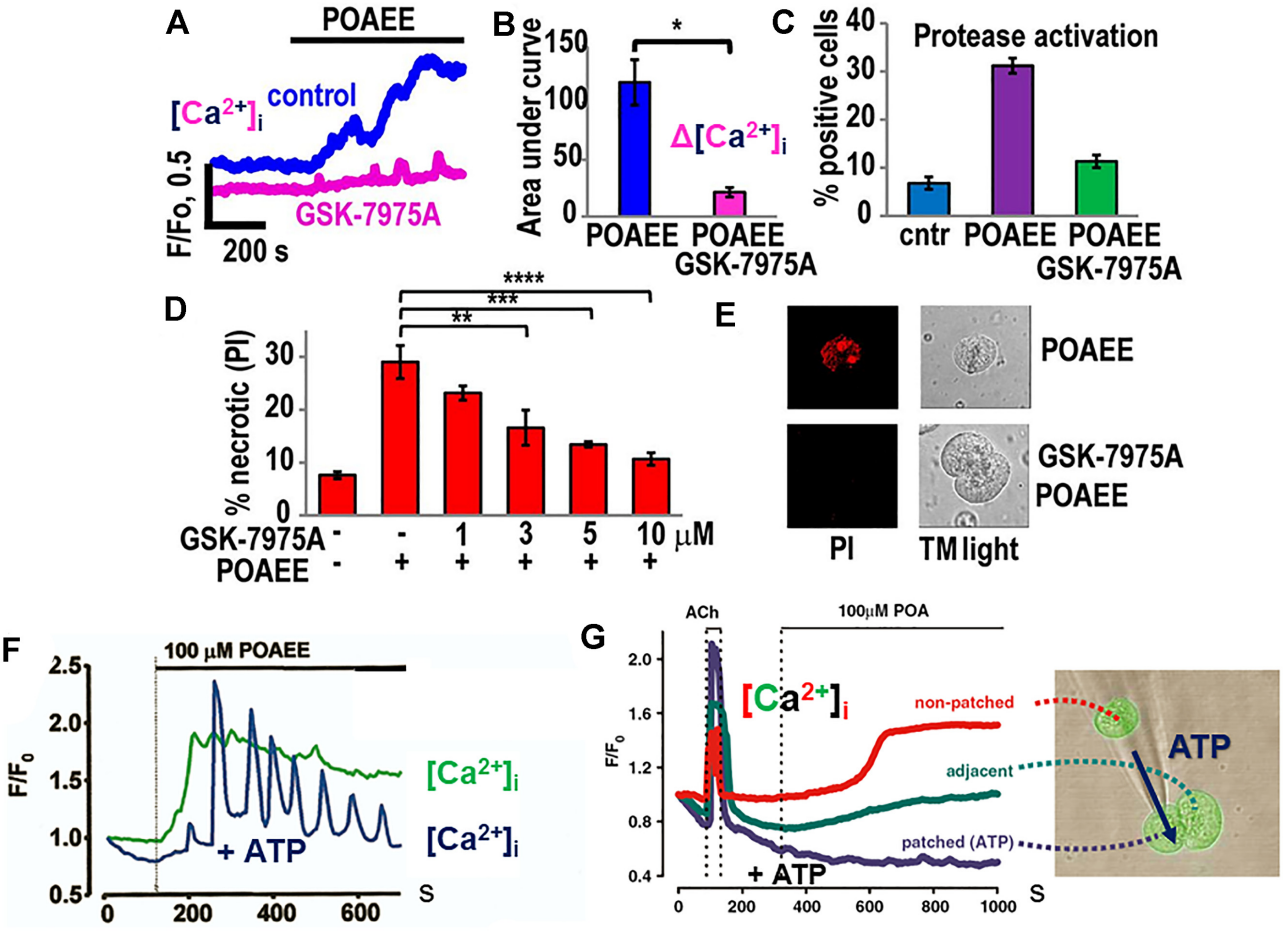
Ca^2+^ signals, protease activity and necrosis following stimulation with the fatty acid ethyl ester POAEE or the fatty acid POA. (A) Ca^2+^ signals generated by POAEE in the absence and presence of the CRAC channel inhibitor GSK-7975A. (B) Quantification of the data represented by the traces in A. (C) GSK-7975-A markedly reduces the POAEE-elicited increase in intracellular protease activity. (D) Concentration-dependent reduction in POAEE-induced necrosis by GSK-7975A. (E) Protection by GSK-7975-A against POAEE-induced uptake of propidium iodide (PI) (necrosis). (F) Typical POAEE-elicited [Ca^2+^]_i_ elevation. When ATP is injected into the cell (see also image in G) the sustained [Ca^2+^]_i_ elevation is transformed into a series of spikes. (G) [Ca^2+^]_i_ elevation elicited by POA. It is much slower than in the case of POAEE stimulation (F) and is abolished when ATP is injected into the cell. ATP can to some extent diffuse into an adjacent cell and in this way partially reduce the POA-elicited [Ca^2+^]_i_ elevation. A–E are adapted from Gerasimenko et al. 2013,^59^ F and G are adapted from Criddle et al. 2006.^66^

The POAEE-elicited prolonged elevated [Ca^2+^]_i_ plateau depended on Ca^2+^ entry through CRAC channels and pharmacological inhibition of the opening of these channels markedly reduced the POAEE-elicited [Ca^2+^]_i_ rise (Figure 10). As expected, the POAEE-elicited toxic Ca^2+^ signal caused intracellular protease activation and led to necrotic cell death. These effects could also be prevented by CRAC channel inhibition (Figure 10). This suggested the possibility of treating AP by selective pharmacological CRAC channel inhibitors.^59^ Such CRAC channel inhibition has been shown to be effective in preventing and treating experimental AP ^59,84,85^ Clinical trials are currently in progress.^86,87^

When we were able to assess changes in mitochondrial and cytosolic ATP levels, it turned out that both POA and POAEE caused reductions in the ATP level, whereas physiological stimulation with CCK elicited a rise.^67^ As seen in Figure 10, the actions of POA and POAEE are somewhat different. Whereas POAEE evokes a relatively fast rise in [Ca^2+^]_i_ (Figure 10F), POA acts more slowly (Figure 10G). Furthermore, intracellular ATP injection prevents POA from causing a Ca^2+^ signal, but only reduces the elevation of [Ca^2+^]_i_ evoked by POAEE (Figure 10F and G). Taking all these data into account,^66,67^ it would appear that POAEE elicits a massive release of Ca^2+^ from internal stores, which in turn causes excessive opening of CRAC channels flooding the cytosol with Ca^2+^. This overloads the mitochondria with Ca^2+^ and opens the MPTP, depolarizing the inner mitochondrial membrane. ATP generation is therefore inhibited. POA does not primarily seem to elicit any Ca^2+^ release from internal stores and may principally act directly on the mitochondria to inhibit ATP production.^66^

The crucial role of Ca^2+^ in the pathophysiology of AP has been confirmed by two other developments. As already mentioned, the major causes of AP are gallstone complications and alcohol abuse, but AP can also occur as the result of a side effect of the standard treatment with asparaginase of acute lymphoblastic leukemia in children.^88^ We were alerted to this by colleagues at the Great Ormond Street Hospital for children in London, who wanted to find out why asparaginase could cause AP. It turned out that it was, again, related to toxic Ca^2+^ signals in the pancreatic acinar cells.^89,90^ Essentially the same effects as those already described for POAEE were observed, namely sustained [Ca^2+^]_i_ elevation, reduction of intracellular ATP and necrosis.^89,90^ However, in the course of these experiments, we did identify a novel aspect. It turned out that glucose metabolism was impaired in AP, not only in the case of asparaginase action, but also in alcohol-related AP.^90^ This appeared to be due to inhibition of hexokinase and the problem could be circumvented by feeding with galactose, which unlike glucose, can be metabolized via the Lelois pathway and therefore does not depend on hexokinase activity.^87,90^

AP can also occur as the result of direct physical manipulation of the pancreas during surgery or due to high pressure in the ductal system, which can happen as a result of blockage of the main pancreatic duct.^65^ Experiments by Roger Liddle and his group at Duke University have shown that this type of AP is also mediated by toxic Ca^2+^ signals, but the ion channels involved seem to be different from what has so far been described in this article. Liddle and his collaborators showed that there are pressure-sensitive Piezo-1 channels in the pancreatic acinar cell membrane and when these open, a small Ca^2+^ inflow occurs. This activates phospholipase A2 which, in turn, opens TRPV4 channels through which the major Ca^2+^ inflow in this case seems to occur.^91,92^

## It Is Not Sufficient to Observe the Acinar Cells; Stellate Cells Also Deserve a Look

The classical work of Hans Chiari classified AP as an autodigestive disease^93^ and numerous articles have since been published presenting strong evidence for trypsinogen activation as a key element in the initiation of AP.^94,95^ It is, however, a problem that antiprotease agents have been shown in numerous clinical trials to have no effect on AP and currently play no role in guidelines concerning AP treatment.^96^ Although intracellular trypsinogen activation undoubtedly plays a significant role in the destruction of the acinar cells that occurs in AP, it does not seem to be important for the progression of the local and systemic inflammation, as demonstrated in a study on knock-out mice without the trypsinogen gene.^96^ This is an important finding because it is the inflammatory response in AP that is by far the most dangerous part of the disease.^87,96^

**Figure 11.**
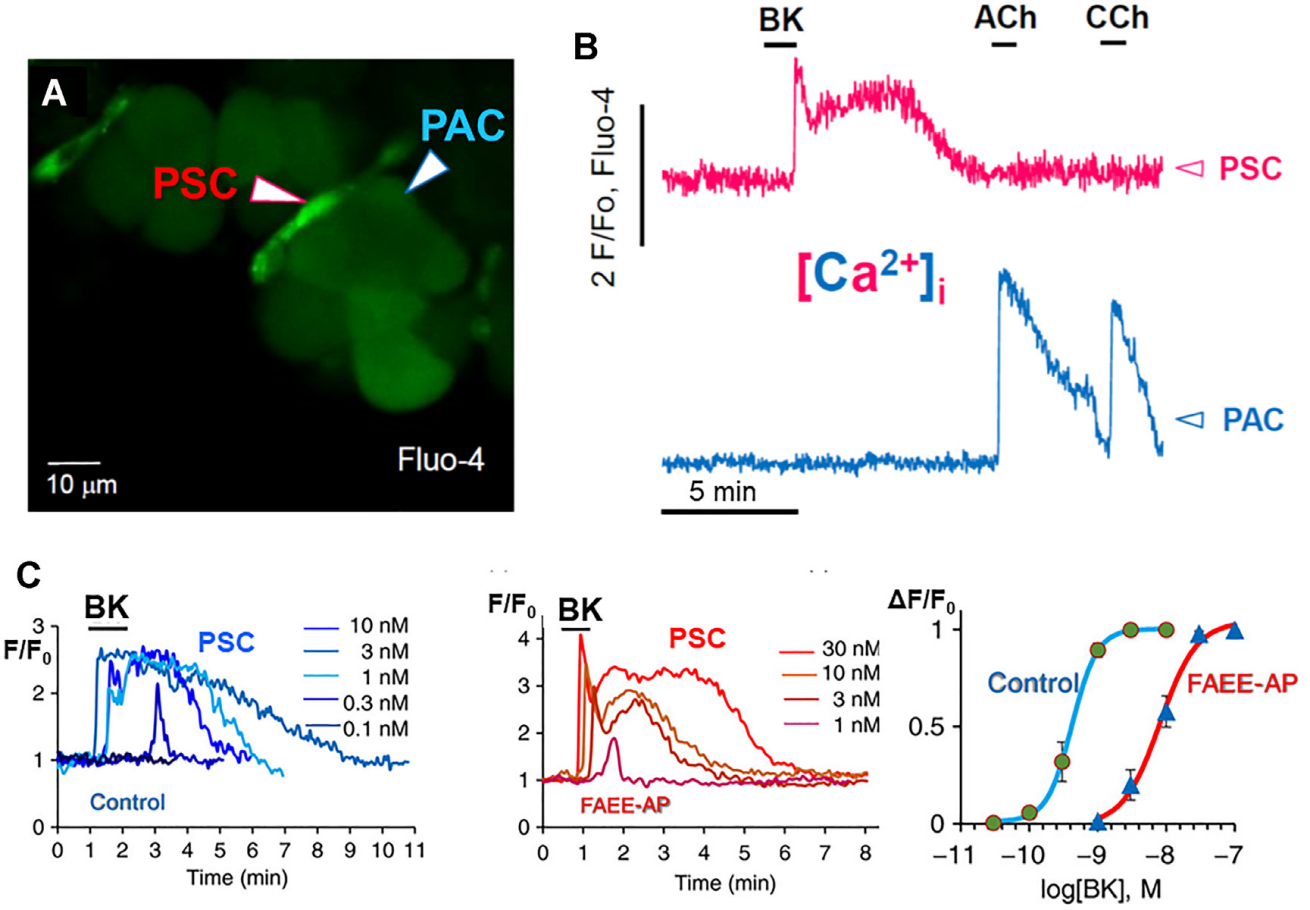
BK elicits Ca^2+^ signals in stellate cells (PSCs) but not in acinar cells (PACs). (A) Fluorescence image (Fluo-4) of one part of an isolated pancreatic lobule. It is seen that small slim cells at the edge of acinar units are lit up indicating that the PSCs take up the fluorescent probe more avidly than the PACs. (B) BK-elicited [Ca^2+^]_i_ rise in the PSC shown in A, which is not transmitted to the neighboring PAC. ACh or Carbachol (CCh) evoke Ca^2+^ signals in the PAC and these elevations are not transmitted to the neighboring PSC. (C) Concentration-response curves for BK-elicited [Ca^2+^]_i_ elevations in normal PSCs and in PSCs from the pancreas of mice 2 d after induction of alcohol-related acute pancreatitis (injection of POA and ethanol—FAEE-AP). A and B are adapted from Gryshchenko et al. 2016 ^97^ and C from Gryshchenko et al. 2018.^48^

It is of course obvious that immune cell function must be critical to the development of the inflammatory response in AP, but until very recently there had been no attempt to visualize directly activity in non-acinar cells in the acinar environment. Morphological studies of the exocrine pancreas have overwhelmingly been focused on the acinar cells. The stellate cells, which are the closest neighbors to the acinar cells, represent a very small volume of the pancreas, can hardly be seen with standard light microscopy and rarely make an appearance in EM pictures. When the pancreas is digested by collagenase, as is routinely done when studying isolated acinar cells or cell clusters, one essentially only sees acinar cells. However, when watching living cells in quasi-intact small pieces of pancreas, and especially with fluorescence microscopy, one can observe that small and thin, but relatively long, cells are located on the basal acinar surface (Figure 11). These are the stellate cells,^97^ which were only described in the pancreas relatively recently.^98^ They are generally regarded as being somewhat similar to the hepatic stellate cells, which were discovered by Carl von Kupfer in 1876, who called them “Sternzellen” (star cells).^99^ These names are clearly related to those of astrocytes (Astron [Greek] meaning star).^100^ Astrocytes, hepatic and pancreatic stellate cells have in common that they were originally regarded as relatively passive in their respective organs. The situation for the astrocytes, in particular, has changed dramatically in recent years and there is now a huge literature on their many and highly important functions.^100^

The pancreatic stellate cells are in fact far from passive. They generate Ca^2+^ signals in response to stimulation with bradykinin (BK), but not with ACh (Figure 11) or CCK.^97^ In spite of the proximity of the stellate cells to the acinar cells, Ca^2+^ signals elicited in the stellate cells are not transmitted to the acinar cells and vice versa (Figure 11A and B). Interestingly, BK has been shown to evoke Ca^2+^ signals in astrocytes and these are also not directly transmitted to neighboring neurons, but cause Ca^2+^-dependent release of glutamate, which in turn elevates [Ca^2+^]_i_ in the neurons.^101^ In the presence of a glutamate receptor antagonist, BK still evoked Ca^2+^ signals in astrocytes, but no longer in adjacent neurons.^101^ The BK concentrations reported to evoke Ca^2+^ signals in astrocytes were much higher (100 nM–1 µm) ^101^ than those required in the pancreatic stellate cells (Figure 11).

BK is a small inflammatory peptide that is split off from the large bradykininogen molecule by the action of the protease kallikrein, which is produced in the acinar cells. Many years ago, Ryan, Moffat and Thompson from Montreal General Hospital proposed that BK could play an important role in the development of AP.^102^ They showed that induction of AP in dogs was associated with a major loss of bradykininogen and generation of BK.^102^ An increased plasma BK level in AP was confirmed many years later^103^ and it was also shown that pharmacological blockade of BK type 2 receptors (the receptors responsible for the BK-elicited Ca^2+^ signals in the stellate cells^97^) inhibited the development of the cellular changes that are characteristic for AP.^103^

Having observed directly the impressive BK-elicited Ca^2+^ signals in the stellate cells (Figure 11), we were interested in pursuing this issue. When describing a new phenomenon, such as the BK-elicited Ca^2+^ signals, it is always useful, at an early stage, to establish the concentration-response relationship (Figure 11C). It is helpful to relate this curve to what is known about the plasma BK concentrations during resting conditions and in AP. A slight elevation of the BK concentration above the resting level (just below 0.1 nM ^87^) is sufficient to evoke a clear Ca^2+^ signal and a near-maximal response occurs at BK concentrations that have been measured during AP (∼1 nM–Figure 11C).^87^ These results are compatible with the hypothesis that BK plays an active role in AP, but do not prove it. However, we did show that BK (type 2) receptor antagonists markedly reduce the extent of necrosis evoked by FAEEs and bile acids.^97^ Furthermore, a couple of days after induction of alcohol-related experimental AP, it was apparent that there had been a desensitization of the response of the stellate cells to BK (Figure 11C). Whereas in the stellate cells from normal mice, BK at 0.1 nM could elicit clear Ca^2+^ signals, a concentration of 1 nM was required in the cells from the AP mice and a near-maximal response could only be evoked by 10–30 nM BK (Figure 11C). In other words, the BK concentration—response curve in the stellate cells from the AP mice had been shifted markedly to the right (Figure 11C). This is most easily explained by BK action, at a continuously high concentration, during the first day of AP, in complete agreement with the original findings of Ryan et al. already mentioned.^102^ The changes in the sensitivity of the stellate cells occurring during the first days of alcohol-related AP are remarkable. There is not only desensitization to BK, but also an important increase in sensitivity to trypsin and thrombin,^97^ which could well be of real pathophysiological significance.

Live imaging showed that Ca^2+^ signals in the stellate cells generate nitric oxide (NO).^104^ It would appear that this has a deleterious effect on the neighboring acinar cells, because pharmacological inhibition of the Ca^2+^-sensitive NO synthase markedly reduced the extent of necrosis evoked by bile.^104^ The mechanism by which NO acts, directly or indirectly, is currently unknown. Overall, the live imaging work on stellate cells in the quasi-intact pancreas has provided compelling evidence for an active role of these cells in early stages of AP, although many mechanistic aspects remain unclear.

The BK-elicited Ca^2+^ signals in the stellate cells occur by a mechanism that is very similar to the one described for the action of ACh on the acinar cells. The initial steep rise in ([Ca^2+^]_i_) is independent of the presence of external Ca^2+^ and therefore due to release from internal stores. However, unlike the action of ACh on the acinar cells, this initial rapid spike-like [Ca^2+^]_i_ rise is always followed by a prolonged elevated [Ca^2+^]_i_ plateau (Figure 11) and this is acutely dependent on external Ca^2+^ as well as being abolished by CRAC channel inhibition.^97^ Thus, CRAC channel inhibition will not only protect acinar cells against toxic Ca^2+^ signal generation, but also reduce Ca^2+^ inflow to the stellate cells.

## Watching Ca^2+^ Signals in Endogenous Immune Cells

**Figure 12.**
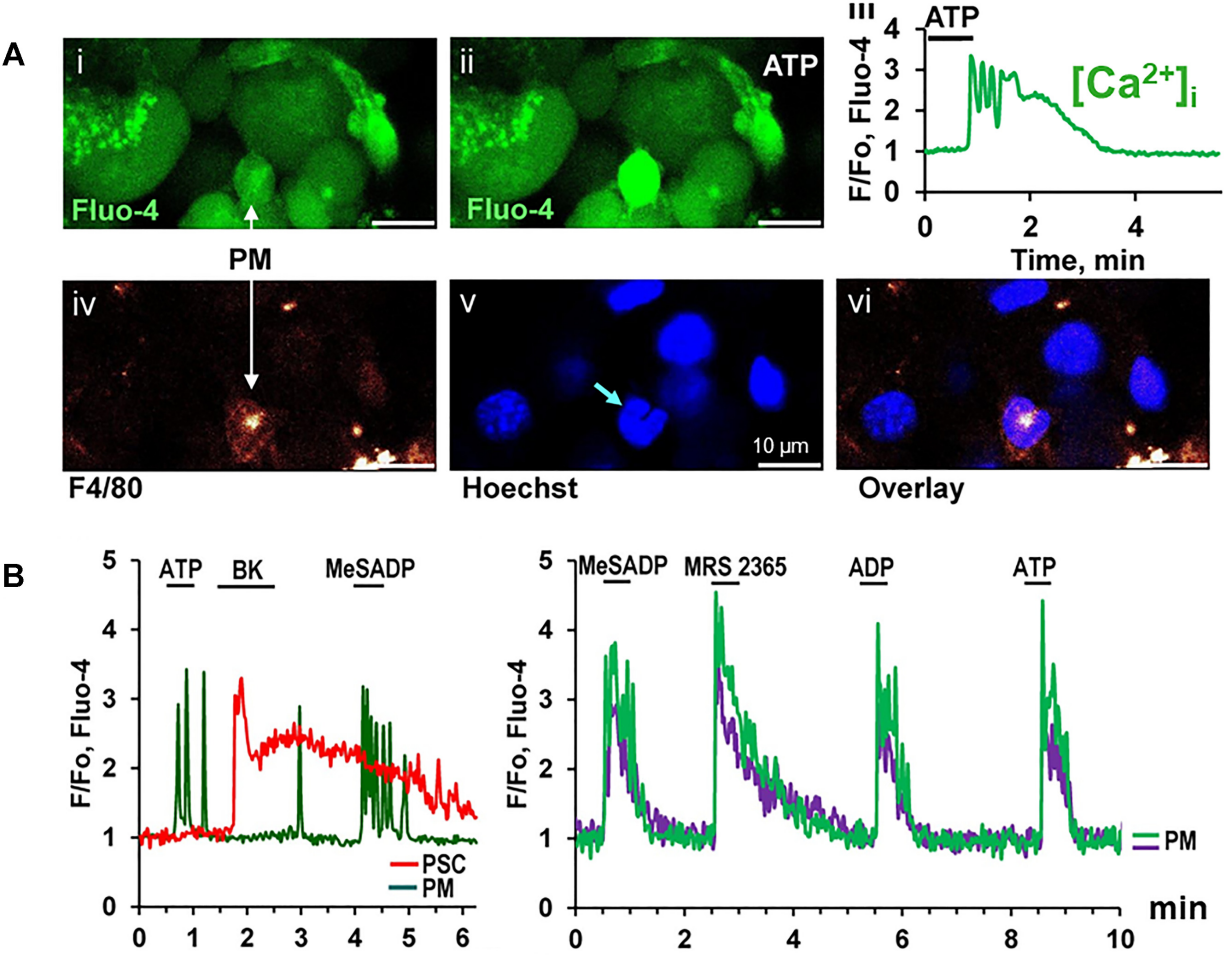
Activation of purinergic receptors on pancreatic macrophages in situ elicit Ca^2+^ signals. (A) ATP elicits Ca^2+^ signal in cell that is subsequently stained with F4/80. (i) Fluo-4 fluorescence picture before stimulation. (ii) ATP has increased [Ca^2+^]_i_. (iii) Time course of ATP-elicited [Ca^2+^]_i_ change. (iv) Staining with F4/80. (v) Nuclear staining with Hoechst 33342. (vi) Overlay of iv and v. (B) ADP and selective agonists of P2Y_1_ and P2Y_13_ receptors have the same effects on [Ca^2+^]_i_ as ATP. See text for further details. Adapted from Gryshchenko et al. 2021.^105^

In the normal pancreas there are few immune cells, but we were nevertheless interested in finding out whether these could be involved in signaling events. We identified a non-acinar cell type that generated repetitive Ca^2+^ spikes when stimulated by either ATP or ADP (Figure 12). Such cells were, at the end of functional experiments, identified as macrophages due to their staining with the fluorescently labeled antibodies F4/80 (Figure 12) or CD11b.^105^ There is a remarkably rich pharmacology of purinergic receptors^106^ and, taking advantage of this knowledge, we could show that the purinergic receptors on the endogenous macrophages belonged to the P2Y_1_ or P2Y_13_ types. Thus MeSADP (a P2Y_1_ and P2Y_13_ agonist) as well as MRS 2365 (a highly selective P2Y_1_ agonist) mimicked the effects of ATP and ADP (Figure 12) and the P2Y_1_ antagonist MRS 2179 and the competitive P2Y_13_ antagonist MRS 2211 blocked the responses to the purinergic agonists.^105^

The number of macrophages in the pancreatic tissue increased markedly after induction of alcohol-related AP in mice. Three days after start of injection of ethanol and POA, the density of macrophages was six times higher than in the normal pancreas, whereas the density of stellate cells was unchanged.^105^ Furthermore, the macrophages became sensitive to IgG stimulation. Although the latency was long (several minutes), IgG elicited substantial repetitive Ca^2+^ spikes, which were longer than the occasional single spike that could occur in macrophages in the normal healthy pancreas.^105^

The Ca^2+^ signals elicited in the pancreatic macrophages by ADP or ATP were due to primary release of Ca^2+^ from intracellular pools followed by Ca^2+^ entry through CRAC channels.^105^ The Ca^2+^ signals in the macrophages were, however, more quickly reduced in the absence of external Ca^2+^ or in the presence of CRAC channel inhibition than, for example, the ACh-elicited Ca^2+^ signals in the acinar cells.^105^ The acinar cells do of course have a very large ER, and therefore a much larger intracellular Ca^2+^ store than most other cell types.

So far, we have no direct evidence concerning the functional consequences of the Ca^2+^ signal generation in the endogenous macrophages in the pancreas. However, based on evidence from immune cells isolated from the blood,^107^ it seems highly likely that the Ca^2+^ signals in the pancreatic macrophages will stimulate secretion of inflammatory cytokines.

Although the majority of the pancreatic macrophages did not display Ca^2+^ signals in response to BK stimulation (Figure 12B), there was a substantial minority (∼40%) that did so, although higher concentrations of the nonapeptide were required than was the case for eliciting Ca^2+^ signals in the stellate cells.^105^ Because BK levels in plasma are elevated in AP, some of the macrophages that invade the pancreas in the initial phase of the disease may also, as is the case for the stellate cells, be further stimulated by BK and in this way participate in both the cytokine and BK storms that are characteristic of severe AP.^87,105^ From a therapeutic perspective, it is important that all Ca^2+^ signals in pancreatic macrophages can be inhibited by pharmacological CRAC channel inhibitors, as is also the case for the acinar and stellate cells.^59,97,105^ The remarkable effectiveness of CRAC channel inhibition in protecting against experimental AP is therefore almost certainly due to actions on all three cell types.^87^

## SARS-CoV-2 and Acute Pancreatitis

**Figure 13.**
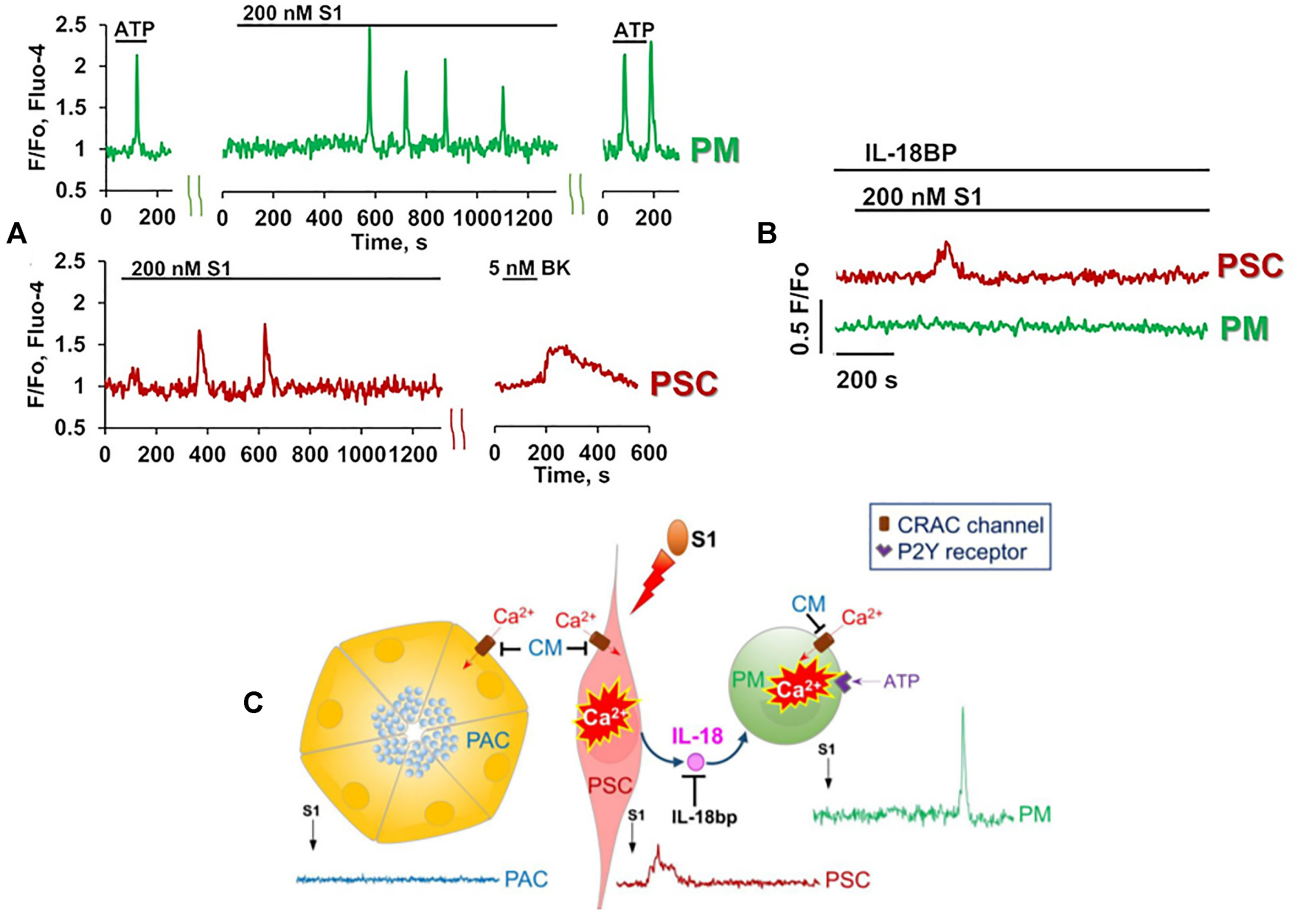
The spike protein of SARS-CoV-2 (S1) evokes Ca^2+^ signals in macrophages (PMs) and stellate cells (PSCs) in situ. (A) S1 evokes Ca^2+^ spikes in an ATP-sensitive pancreatic macrophage (PM—green trace) and in a BK-sensitive PSC (red trace). (B) Interleukin-18 binding protein (IL-18BP) prevents S1 from eliciting Ca^2+^ signal in PM (green trace), but not in adjacent PSC (red trace). (C) Model concept of S1-induced Ca^2+^ signaling in macrophages. CM means CM4620 (a CRAC channel inhibitor). Adapted from Gerasimenko et al. 2022.^113^

SARS-CoV-2 entry into cells occurs via binding of its S protein subunit to ACE2 receptors allowing endocytic virus uptake.^108,109^ The virus has been shown to enter many different cell types in the body. These include cells in the respiratory tract, the nervous system and the gastrointestinal tract.^109–112^ There are some similarities between severe AP and severe COVID-19 as, in both cases, ARDS (Acute Respiratory Distress Syndrome) plays an important role.^87^ The cytokine and BK storms cause massive inflammation, including severe vasodilatation and extravasation, impeding oxygen transfer from the alveoli to the blood. We were therefore interested in testing whether SARS-CoV-2 might act directly on macrophages and/or stellate cells in the pancreatic tissue. As seen in Figure 13, the SARS-CoV-2 spike protein elicited Ca^2+^ spikes in both endogenous macrophages and stellate cells, whereas no effects were observed in the acinar cells.^113^ All Ca^2+^ signals could be blocked by CRAC channel inhibition.^113^

It was noticeable that the latency for Ca^2+^ signal generation in the macrophages was longer than in the stellate cells (Figure 13). This might suggest that the Ca^2+^ signals in the macrophages were generated secondarily to events in the stellate cells. It turned out that an interleukin binding protein could prevent the SARS-CoV-2 spike protein from generating Ca^2+^ spikes in the macrophages, but not in the stellate cells (Figure 13).

## The Stellate Cells Are Important

More work clearly needs to be carried out on the mechanism of action of SARS-CoV-2 on pancreatic stellate cells, but at least the experiments conducted so far (Figure 13) highlight the importance and central role of the stellate cells in pancreatic disease. Very recent work further highlights this central role. It turns out that the combination of POA and ethanol, generating POAEE inside pancreatic cells, in addition to eliciting the large and sustained Ca^2+^ signals in the acinar cells already described (Figure 10), is capable of inducing such toxic Ca^2+^ signals in the stellate cells.^114^ Thus, toxic Ca^2+^ signals in the stellate cells may not necessarily be secondary to toxic Ca^2+^ signals generated in the acinar cells, but can also occur as a primary event. The stellate cells may therefore play an even bigger role in the development of AP than hitherto thought^87^. Furthermore, it has now been shown that the pressure-sensitive Piezo-1 channels are functional also in stellate cells.^115^ The mechanism, already described in acinar cells, whereby physical pressure can elicit small Ca^2+^ signals mediated via Piezo-1 channels that subsequently lead to opening of TRPV4 channels, generating a more substantial Ca^2+^ influx, also appears to operate in the stellate cells.^115^ Physical pressure could therefore evoke Ca^2+^ signals in both acinar and stellate cells.

## Conclusion

Acute observations in real time of signaling and secretion events in identified cell types in the exocrine pancreas have provided valuable information about physiological and pathophysiological processes in this gland. The mechanisms underlying the principal functions of the acinar cells have been particularly well investigated and are now reasonably well understood, whereas studies of the stellate and immune cells are still in their infancy. Future studies of pancreatic pathophysiology will almost certainly depend on direct imaging studies of these cells and their interactions with each other in the intact pancreas in situ. With the recent spectacular developments in in vivo imaging technology,^116,117^ we can expect much enlightenment in the coming years.

## Data Availability

In this review article there are no new data presented
